# Long-term survival of patients with advanced melanoma treated with BRAF-MEK inhibitors

**DOI:** 10.1097/CMR.0000000000000832

**Published:** 2022-09-05

**Authors:** Rawa K. Ismail, Karijn P.M. Suijkerbuijk, Anthonius de Boer, Maaike van Dartel, Doranne L. Hilarius, A.M.G. Pasmooij, Michiel C.T. van Zeijl, Maureen J.B. Aarts, Franchette W.P.J. van den Berkmortel, Christian U. Blank, Marye J. Boers-Sonderen, Jan W.B. de Groot, John B.A.G. Haanen, Geke A.P. Hospers, Ellen Kapiteijn, Djura Piersma, Rozemarijn S. van Rijn, Astrid A.M. van der Veldt, Art Vreugdenhil, Hans Westgeest, Alfons J. van den Eertwegh, Michel W.J.M. Wouters

**Affiliations:** a Dutch Institute for Clinical Auditing, Leiden; b Division of Pharmacoepidemiology and Clinical Pharmacology, Utrecht Institute for Pharmaceutical Sciences, Utrecht; c Medicines Evaluation Board, Utrecht; d Department of Medical Oncology, University Medical Centre Utrecht, Utrecht; e Department of Pharmacy, Rode Kruis Ziekenhuis, Beverwijk; f Department of Medical Oncology, Leiden University Medical Centre, Leiden; g Department of Medical Oncology, Grow School for Oncology and Developmental Biology Maastricht University Medical Centre, Maastricht; h Department of Medical Oncology, Zuyderland Medical Centre Sittard, Sittard-Geleen; i Department of Medical Oncology and Immunology, Netherlands Cancer Institute, Amsterdam; j Department of Medical Oncology, Radboud University Medical Centre, Nijmegen; k Department of Medical Oncology, Isala, Zwolle; l Department of Medical Oncology, University Medical Centre Groningen, Groningen; m Department of Internal Medicine, Medisch Spectrum Twente, Enschede; n Department of Internal Medicine, Medical Centre Leeuwarden, Leeuwarden; o Department of Medical Oncology and Radiology & Nuclear Medicine, Erasmus Medical Centre, Rotterdam; p Department of Internal Medicine, Maxima Medical Centre, Eindhoven; q Department of Internal Medicine, Amphia Hospital, Breda; r Department of Medical Oncology, Amsterdam University Medical Center, Amsterdam; s Department of Surgical Oncology, Netherlands Cancer Institute, Plesmanlaan, Amsterdam; t Department of Biomedical Data Sciences, Leiden University Medical Centre, Leiden, The Netherlands

**Keywords:** advanced melanoma, BRAF-MEK inhibitors, survival rates

## Abstract

Recent results of patients with advanced melanoma treated with first-line BRAF-MEK inhibitors in clinical trials showed 5-year survival in one-third of patients with a median overall survival (OS) of more than 2 years. This study aimed to investigate these patients’ real-world survival and identify the characteristics of long-term survivors.

The study population consisted of patients with advanced cutaneous melanoma with a BRAF-V600 mutated tumor who were treated with first-line BRAF-MEK inhibitors between 2013 and 2017. Long-term survival was defined as a minimum OS of 2 years from start therapy.

The median progression-free survival (mPFS) and median OS (mOS) of real-world patients (*n* = 435) were respectively 8.0 (95% CI, 6.8–9.4) and 11.7 (95% CI, 10.3–13.5) months. Two-year survival was reached by 28% of the patients, 22% reached 3-year survival and 19% reached 4-year survival. Real-world patients often had brain metastases (41%), stage IV M1c disease (87%), ECOG PS ≥2 (21%), ≥3 organ sites (62%) and elevated LDH of ≥250 U/I (49%). Trial-eligible real-world patients had an mOS of 17.9 months. Patients surviving more than 2 years (*n* = 116) more often had an ECOG PS ≤1 (83%), normal LDH (60%), no brain metastases (60%), no liver metastases (63%) and <3 organ sites (60%).

Long-term survival of real-world patients treated with first-line BRAF-MEK inhibitors is significantly lower than that of trial patients, which is probably explained by poorer baseline characteristics of patients treated in daily practice. Long-term survivors generally had more favorable characteristics with regard to age, LDH level and metastatic sites, compared to patients not reaching long-term survival.

## Introduction

The systemic treatment landscape for advanced (i.e. unresectable stage IIIc or IV) melanoma patients has dramatically changed in recent years with the introduction of immunotherapies (CTLA-4 and PD-1 inhibitors) and targeted therapies (BRAF- and MEK-inhibitors) [1]. In 40–50% of the patients with advanced melanoma, *BRAF* gene mutations are present, leading to the continued activation of the mitogen-activated protein kinase (MAPK) signaling pathway and increased cell growth and proliferation [2]. Targeted therapies inhibit BRAF- and MEK-proteins in the MAPK signaling pathway. Treatment of BRAF mutated patients with these BRAF-MEK inhibitors led to major improvements in patient outcomes regarding response and survival [3].

In 2012, BRAF inhibitor (BRAFi) vemurafenib was authorized by the European Medicines Agency (EMA), followed by BRAF-inhibitors dabrafenib in 2013 and encorafenib in 2018. The addition of MEK-inhibitors (cobimetinib, trametinib and binimetinib) to BRAF-inhibitors further improved clinical outcomes over monotherapy due to the dual blockade of proteins in the MAPK signaling pathway. These results led to the approval of combined targeted therapy with BRAF-MEK inhibitors as standard therapy in patients with advanced melanoma [3,4].

Recently, updated results from the phase III clinical trials were published demonstrating long-term survival outcomes of patients treated with BRAF-MEK inhibitors. The COMBI-d and COMBI-v trial, including patients treated with dabrafenib/trametinib, showed a 5-year survival rate of 34% [95% confidence interval (CI), 30–38%] and a 5-year progression-free survival (PFS) rate of 19% (95% CI, 15–22%) [5]. Two- and 3-year survival rates were 52% (95% CI, 45–59%) and 44% (95% CI, 36–51%), respectively [4]. Extended 5-year follow-up results of patients treated in the BRIM-7 trial with vemurafenib/cobimetinib showed a 5-year survival of 39% (95% CI, 26–52%) in BRAF inhibitor-naïve patients [6]. Patients treated with encorafenib/binimetinib in the COLUMBUS trial had a 5-year overall survival (OS) rate of 35% (95% CI, 28–42%) [7]. Considering the heterogenic patient population and the uncontrolled setting in daily practice [8,9], it is of major relevance to know how these data from the clinical trials translate into benefits in real-world patients.

Information on which patients are likely to benefit long-term and which treatment strategies are best used to achieve long-term survival is essential. This information can be used in daily clinical practice to support treatment decisions and help to set realistic expectations for individual patients. This study aimed to investigate the real-world survival of patients with advanced melanoma treated with BRAF-MEK inhibitors and identify the patient, tumor and treatment characteristics of those who derive long-term benefits.

## Patients and methods

### Data source

Data were retrieved from the Dutch Melanoma Treatment Registry (DMTR). The DMTR is a prospective population-based registry with baseline patient, tumor, treatment characteristics and clinical outcomes of all patients with advanced melanoma in the Netherlands [10]. In compliance with Dutch regulations, the DMTR was approved by a medical ethical committee (METC Leiden University Medical Center, 2013) and is not considered subject to the Medical Research Involving Human Subjects Act. The dataset cutoff date was 15 July 2021.

### Patients

All advanced cutaneous melanoma patients with a BRAF-V600 mutated tumor who received first-line BRAF-MEK inhibitors between 1 January 2013 and 31 December 2017, were included. Uveal and mucosal melanoma patients and patients under 18 years were excluded from this study. Patients treated with induction BRAF-MEK therapy were also excluded. This was defined as short therapy (<3 months) with BRAF-MEK inhibitors followed by treatment with checkpoint inhibitors without any signs of progression. Long-term survival was defined as a minimum OS of 2 years from start therapy.

A subanalysis on trial-eligible patients was performed, using the eligibility criteria of the COMBI-D trial. Eligible patients were defined as patients with Eastern Cooperative Oncology Group Performance Score (ECOG PS) ≤1, no brain metastases, BRAF V600E or V600K mutation, and no previous surgery, treated with first-line BRAF-MEK inhibitors.

### Data analysis

Descriptive statistics were used to analyze baseline patient- and disease characteristics at diagnosis. Baseline characteristics of real-world patients treated with first-line BRAF-MEK inhibitors were compared to patients treated in phase III clinical trials. The PFS and OS were estimated using the Kaplan–Meier method with a corresponding two-sided 95% CI. PFS was calculated from the start of BRAF-MEK inhibitors to the date of progressive disease or death. OS was calculated from the start of BRAF-MEK inhibitors to the date of death from any cause or last follow-up. A multivariable Cox proportional hazards regression model was used to estimate the association of prognostic factors with survival. Factors included in this model were age, sex, disease stage calculated with the American Joint Committee on Cancer 7th edition, ECOG PS, lactate dehydrogenase (LDH), the number of organ sites metastasized, brain metastases and liver metastases. Only complete cases were included in the model. Treatment characteristics of long-term survivors were analyzed with descriptive statistics and visualized by a Sankey diagram. ‘Other’ treatment was defined as treatment registered as other, chemotherapy or trial treatment in the DMTR. The treatment duration of BRAF-MEK inhibitors was calculated using the start- and stop date of the BRAF-inhibitor. If the date of discontinuation was missing, the date of the last contact was used. The median follow-up times for PFS and OS were calculated with the reverse Kaplan–Meier method [11].

Data handling and statistical analyses were performed using the R software system for statistical computing (version 4.1.0.; packages tidyr, ggplot2, tableone, ggthemes, stringr, forestmodel, car, survival, survminer, ggalluvial and easyalluvial) [12].

## Results

### Patient characteristics of the study population

**Fig. 1:**
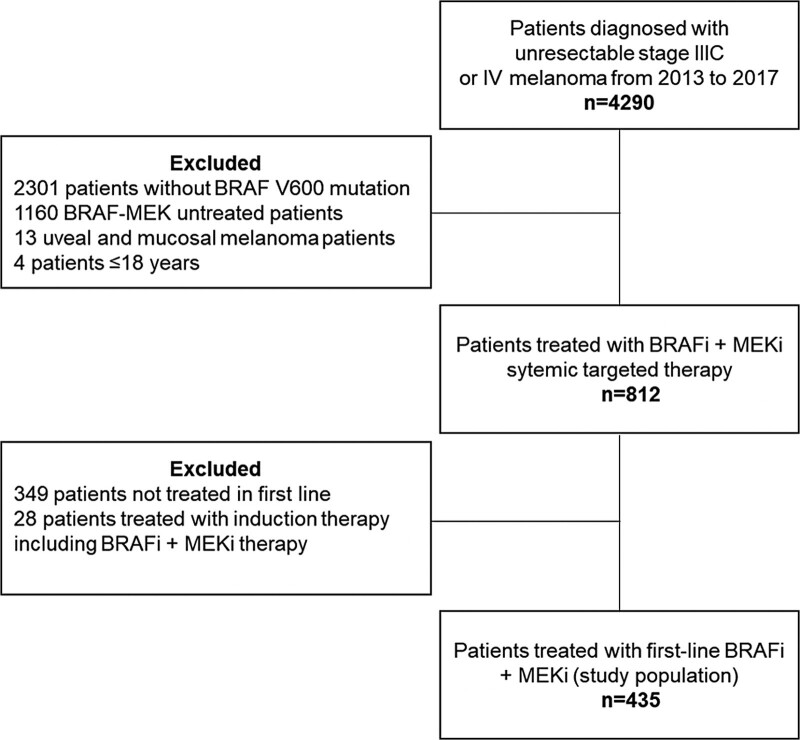
Flowchart of the study population included in this study.

Of the 4290 patients diagnosed with irresectable melanoma between 2013 and 2017, a total of 435 patients received BRAF-MEK-inhibitors in the first line (Fig. 1). The differences in patient- and tumor characteristics between patients treated with BRAF-MEK inhibitors in the real-world study population and phase III trials are shown in Table 1. Real-world patients were older (median age 59 vs. 55–56 years), more often had an ECOG PS of ≥2 (21 vs. <1%), M1c disease (87 vs. 59–63%) and metastases in ≥3 organ sites (62 vs. 50%) than trial patients. Normal LDH levels were less often found in real-world patients than in trial patients (48 vs. 54–66%). A total of 243 (55%) real-world patients received subsequent anticancer therapy after the first-line BRAF-MEK.

**Table 1:** Patients treated in the phase-III study versus real-world patient cohort

	Combi-v trial (dabrafenib/trametinib) [4]	CoBRIM trial (vemurafenib/cobimetinib) [17]	Real-world patients treated with BRAF-MEK inhibitors
Patients; *n*	352	247	435
Median age; years (range)	55 (18–91)	56 (23–88)	59 (19–91)
Male patients; *n* (%)	208 (59)	146 (59)	229 (53)
ECOG PS; *n* (%)			
0	248/350 (71)	184/243 (76)	151 (35)
1	102/350 (29)	58/243 (24)	151 (35)
≥2	0	1/243 (<1)	90 (21)
Unknown	0		43 (10)
Stage (AJCC 7th); *n* (%)			
IVM1c	221/351 (63)	146 (59)	378 (87)
IIIc, IVM1a, IVM1b	130/351 (37)	101 (41)	57 (13)
Metastasis stage; *n* (%)			
M0	14/351 (4)	21 (9)	31 (7)
M1a	55/351 (16)	40 (16)	14 (3)
M1b	61/351 (17)	40 (16)	12 (3)
M1c	221/351 (63)	146 (59)	378 (87)
Number of disease sites; *n* (%)			
<3	177/351 (50)	NA	164 (38)
≥3	174/351 (50)	NA	271 (62)
Baseline LDH; *n* (%)			
Above ULN	118/351 (34)	112/242 (46)	215 (49)
ULN or less	233/351 (66)	130/242 (54)	209 (48)
Unknown	–	_	11 (3)
BRAF mutation; *n* (%)			
V600E	312/346 (90)	170 (69)	361 (83)
V600K	34/346 (10)	24 (10)	61 (14)
Not evaluated	0	53 (21)	0
Other V600	0	0	13 (3)
Previous immunotherapy; *n* (%)	61 (17)	NA	0
Type of BRAF-MEK inhibitors; *n* (%)			
Dabrafenib + trametinib	352 (100)	0	372 (86)
Vemurafenib + cobimetinib	0	247 (100)	63 (14)
Enorafenib + binimetinib	0	0	0 (0)
Other	0	0	0 (0)

AJCC, American Joint Committee on Cancer; ECOG PS, Eastern Cooperative Oncology Group Performance Score; LDH, lactate dehydrogenase.

### Progression-free survival and overall survival

**Fig. 2:**
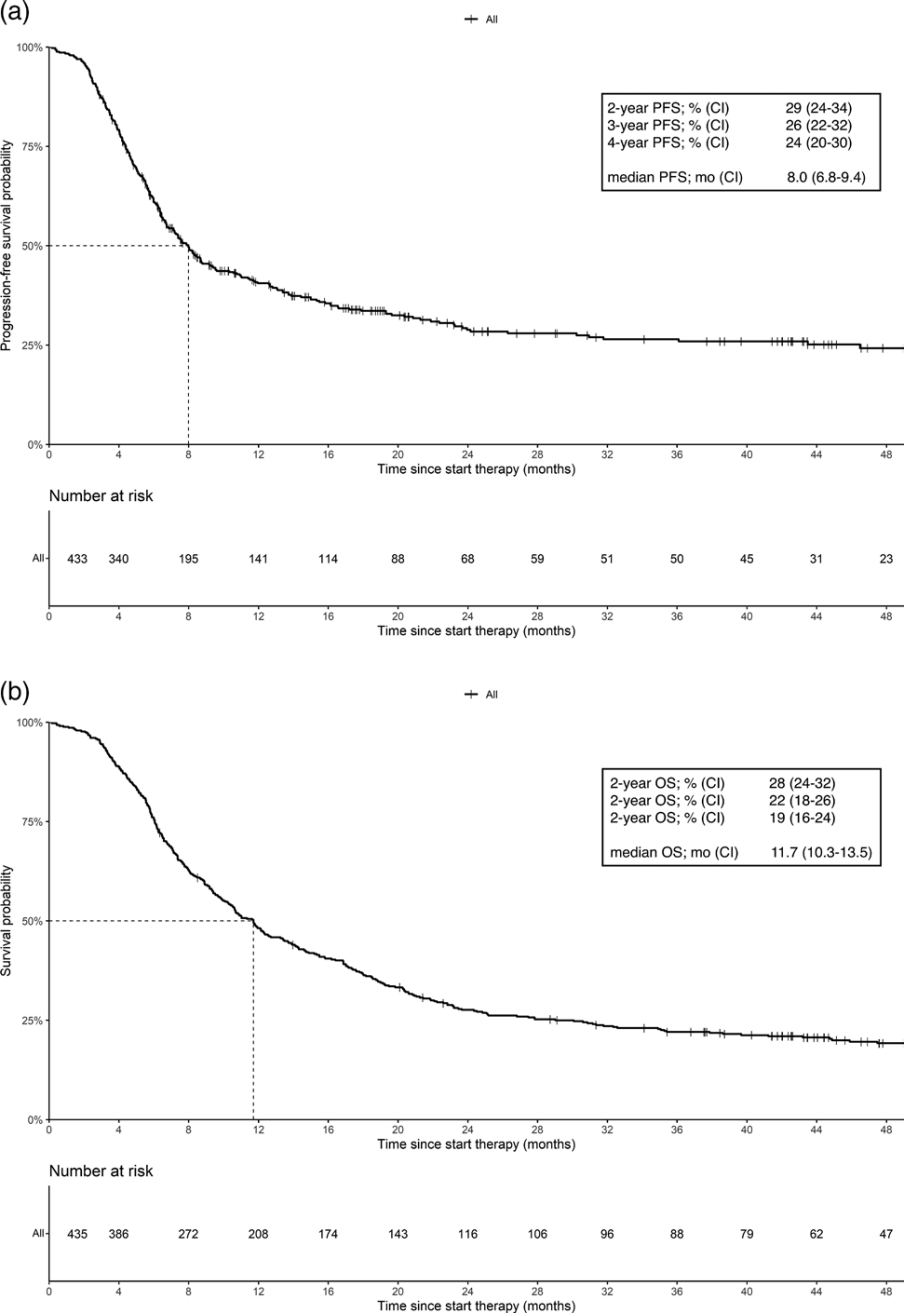
Kaplan-Meier estimates of median, 2-, 3-, and 4-year progression-free survival (a) and median, 2-, 3-, and 4-year overall survival (b) of patients with advanced melanoma treated with first-line BRAF-MEK inhibitors.

The median PFS (mPFS), median OS (mOS) and 2-, 3- and 4-year PFS and OS rates of patients treated with first-line BRAF-MEK inhibitors are shown in Fig. 2a and b. The mPFS and mOS were 8.0 (95% CI, 6.8–9.4) and 11.7 (95% CI, 10.3–13.5) months, respectively. The median follow-up time for PFS was 27.8 (95% CI, 22.8–39.7) and for OS was 51.9 (95% CI, 47.6–55.7) months.

### Best overall response

**Fig. 3:**
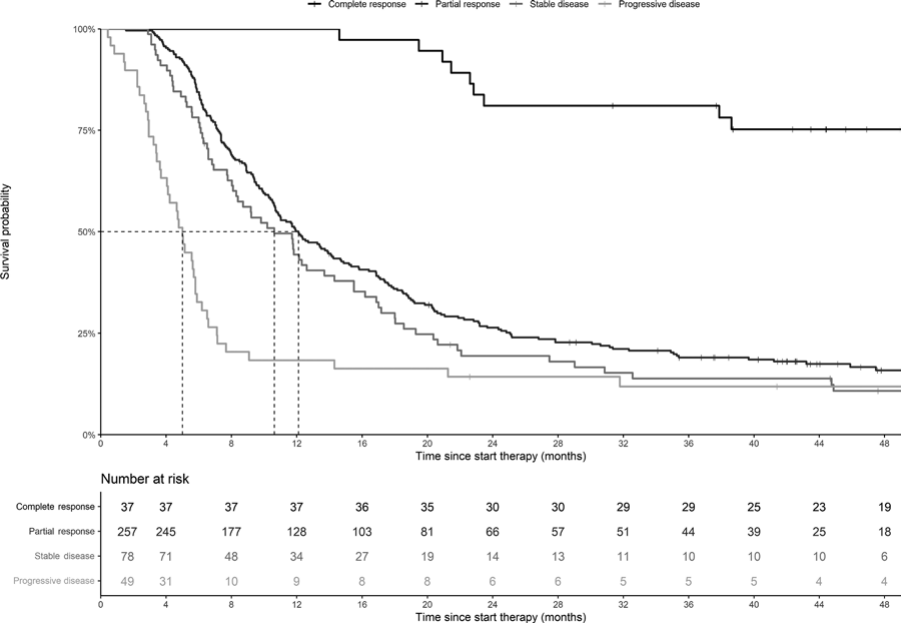
Kaplan–Meier estimates of median overall survival according to the best overall response rate in patients with advanced melanoma treated with first-line BRAF-MEK inhibitors. Fourteen patients were not evaluable for response.

Complete response (CR) occurred in 37 (9%) patients treated with first-line BRAF-MEK inhibitors. Among patients with a CR, the 2-year and 3-year OS were 81% (95% CI, 69–95) and the 4-year OS was 75% (95% CI, 63–91%) (Fig. 3). In patients with a CR who reached 4-year OS (*n* = 19), 53% received no subsequent therapy and 42% received subsequent immunotherapy. Of all patients with CR, 14 (38%) had one or more poor characteristics (brain metastases, ECOG PS ≥2, elevated LDH or ≥3 organ sites) at baseline. Patients with a partial response had a 2-year survival rate of 26% (95% CI, 22–32%). This was 19% (95% CI, 12–31%) for stable disease (Fig. 3). Forty-nine (11%) patients had progressive disease, with an mOS of 5.0 (95% CI, 4.1–5.9) months, and a 2-year OS of 14% (95% CI, 7–28%). Poor baseline characteristics were present in 94% of the patients with progressive disease. The PFS based on the best overall response rate (BORR) is presented in Supplementary Material 2, Supplemental digital content 1, http://links.lww.com/MR/A296.

### Ineligibility

**Fig. 4:**
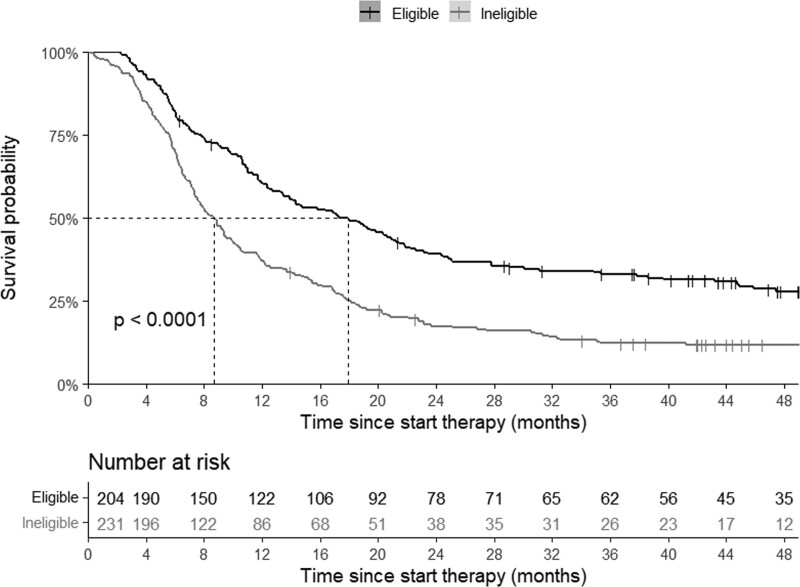
Kaplan-Meier estimates of median, 2-, 3-, and 4-year overall survival of real-world patients with advanced melanoma with characteristics according to trial eligibility criteria, treated with first-line BRAF-MEK inhibitors.

A total of 53% of the patients treated with first-line BRAF-MEK inhibitors was considered ineligible for phase III trial participation. In 77% of the ineligible patients, brain metastases were present (70% were symptomatic and 30% were asymptomatic), and 39% had an ECOG PS ≥2. Patients who would be considered eligible (*n* = 204) had an mOS of 17.9 (95% CI, 13.9–21.4) months, compared to 8.7 (95% CI, 7.5–10.0) months in ineligible patients (*n* = 231) (Fig. 4). The baseline patient- and tumor characteristics of ineligible and eligible patients are shown in Supplementary Material 3, Supplemental digital content 1, http://links.lww.com/MR/A296.

### Patient- and tumor characteristics of long-term survivors

Factors associated with improved survival in the real-world population were age <70 years, LDH <500 U/I, no symptomatic brain metastases and <3 organ sites with metastases (Supplementary Material 1, Supplemental digital content 1, http://links.lww.com/MR/A296).

Baseline characteristics of long-term survivors on first-line BRAF-MEK inhibitors were favorable compared to patients not reaching 2-year OS (Table 2). These long-term survivors more often had an ECOG PS ≤1 (83 vs. 65%), normal LDH (60 vs. 44%), and less often had a highly elevated LDH of ≥500 U/L (3 vs. 23%), stage IVM1c disease (70 vs. 93%) or metastases in ≥3 organ sites (41 vs. 70%). Furthermore, long-term survivors more often did not have brain metastases (60 vs. 47%) and liver metastases (79 vs. 56%) (Table 2). These favorable prognostic factors were even more pronounced in patients who survived more than 3 or 4 years. The proportion of patients experiencing a CR increased when comparing the cohorts of 2-year (26%), 3-year (33%), 4-year (40%) and 5-year (36%) long-term survivors. Normal LDH levels increased from 60% (2-year survival) to 71% (5-year survival) and an ECOG PS ≥2 decreased from 12% to 0% (Table 2). Patients with a minimal 4-year PFS (*n* = 23) generally had favorable characteristics. Still, 4% had an ECOG PS ≥2, one patient (4%) had an LDH level of ≥500 U/L and 13% had brain metastases at baseline.

**Table 2:** Patient-, tumor- and treatment characteristics of patients treated with first-line BRAF-MEK inhibitors not reaching long-term survival versus 2-,3-,4- and 5-year survivors

	Patients surviving <2 years	Patients surviving >2 years	Patients surviving >3 years	Patients surviving >4 years	Patients surviving >5 years
Patients; *n*	319	116	88	47	14
Median age, year (range)	59 (19–88)	58 (27, 91)	58 (27–91)	57 (34–86)	51 (36–84)
Gender; *n* (%)					
Male	173 (54.2)	56 (48.3)	44 (50.0)	18 (38.3)	6 (42.9)
Female	146 (45.8)	60 (51.7)	44 (50.0)	29 (61.7)	8 (57.1)
ECOG PS; *n* (%)					
0	96 (30.1)	55 (47.4)	43 (48.9)	29 (61.7)	11 (78.6)
1	110 (34.5)	41 (35.3)	33 (37.5)	12 (25.5)	2 (14.3)
≥2	76 (23.8)	14 (12.1)	9 (10.2)	3 (6.4)	0 (0.0)
Unknown	37 (11.6)	6 (5.2)	3 (3.4)	3 (6.4)	1 (7.1)
LDH level U/l; n (%)					
Normal	140 (43.9)	69 (59.5)	54 (61.4)	30 (63.8)	10 (71.4)
250–500	98 (30.7)	39 (33.6)	29 (33.0)	14 (29.8)	3 (21.4)
>500	74 (23.2)	4 (3.4)	2 (2.3)	1 (2.1)	0 (0.0)
Not determined	7 (2.2)	4 (3.4)	3 (3.4)	2 (4.3)	1 (7.1)
Stage (AJCC 7th); *n* (%)					
IIIc	12 (3.8)	19 (16.4)	16 (18.2)	9 (19.1)	3 (21.4)
IV-M1a	5 (1.6)	9 (7.8)	9 (10.2)	5 (10.6)	2 (14.3)
IV-M1b	5 (1.6)	7 (6.0)	5 (5.7)	4 (8.5)	1 (7.1)
IV-M1c	297 (93.1)	81 (69.8)	58 (65.9)	29 (61.7)	8 (57.1)
Metastasis in ≥3 organ sites; *n* (%)	224 (70.2)	47 (40.5)	32 (36.4)	15 (31.9)	4 (28.6)
Brain metastasis; *n* (%)					
No	149 (46.7)	70 (60.3)	54 (61.4)	30 (63.8)	10 (71.4)
Yes, asymptomatic	44 (13.8)	10 (8.6)	6 (6.8)	4 (8.5)	1 (7.1)
Yes, symptomatic	109 (34.2)	15 (12.9)	11 (12.5)	4 (8.5)	0 (0.0)
Unknown	17 (5.3)	21 (18.1)	17 (19.3)	9 (19.1)	3 (21.4)
Liver metastasis; *n* (%)	139 (43.6)	24 (20.7)	16 (18.2)	7 (14.9)	2 (14.3)
Best overall response; *n* (%)					
Complete response	7 (2.2)	30 (25.9)	29 (33.0)	19 (40.4)	5 (35.7)
Partial response	191 (59.9)	66 (56.9)	44 (50.0)	18 (38.3)	6 (42.9)
Stable disease	64 (20.1)	14 (12.1)	10 (11.4)	6 (12.8)	3 (21.4)
Progressive disease	43 (13.5)	6 (5.2)	5 (5.7)	4 (8.5)	0 (0.0)
LTFU	14 (4.4)	0 (0.0)	0 (0.0)	0 (0.0)	0 (0.0)
BRAF mutation; *n* (%)					
V600E	269 (84.3)	92 (79.3)	68 (77.3)	37 (78.7)	12 (85.7)
V600K	43 (13.5)	18 (15.5)	15 (17.0)	7 (14.9)	2 (14.3)
Other	7 (2.2)	6 (5.2)	5 (5.7)	3 (6.4)	0 (0.0)
Subsequent therapy after first-line BRAF-MEK; n (%)					
No	158 (49.5)	34 (29.3)	27 (30.7)	16 (34.0)	6 (42.9)
Immunotherapy	151 (47.3)	75 (64.7)	57 (64.8)	28 (59.6)	8 (57.1)
Other	9 (2.8)	7 (6.0)	4 (4.5)	3 (6.4)	0 (0.0)

AJCC, American Joint Committee on Cancer; ECOG PS, Eastern Cooperative Oncology Group Performance Score; LDH, lactate dehydrogenase.

### Treatment characteristics of long-term survivors

In 34 (29%) long-term survivors (>2-year OS), no other subsequent therapy was used. Five (15%) of these patients were treated until the last contact date. These five patients had a median age of 49 years, ECOG PS ≤1, normal LDH-level (80%) and no brain metastases. Subsequent immunotherapy was given to 65% of the long-term survivors. This consisted of anti-PD1 therapy (47%), ipilimumab and nivolumab combination therapy (44%) and ipilimumab monotherapy (9%). A third-line treatment was given to 45% of the patients (Supplementary Material 4, Supplemental digital content 1, http://links.lww.com/MR/A296). Rechallenge with third-line BRAF-MEK inhibitors after second-line immunotherapy treatment (*n* = 64) occurred in 21 patients (33%). Eight patients were treated with third-line immunotherapy after second-line immunotherapy (13%). In patients reaching 4-year OS, 34% received no subsequent treatment and 60% immunotherapy.

The median treatment duration of long-term survivors treated with first-line BRAF-MEK inhibitors was 18.2 months. This was 15.0 months for 3-year and 15.8 months for 4-year survivors. The 34 patients who survived >2 years and received no subsequent therapy had a median treatment duration of 16.2 (IQR 3.7–29.9) months. Long-term survivors treated with subsequent immunotherapy (*n* = 75) received first-line BRAF-MEK inhibitors for 18.8 (IQR 5.8–30.3) months.

## Discussion

This population-based study shows that real-world patients treated with BRAF-MEK inhibitors have poorer survival than trial patients, which is likely related to poorer baseline characteristics such as higher age, poorer ECOG PS, higher LDH, more organ sites with metastases and metastases at locations with known poorer prognosis (brains and liver). Of all patients treated with first-line BRAF-MEK inhibitors in the real world, 28% reached 2-year, 22% 3-year and 19% 4-year survival. Previous real-world studies included smaller cohorts than described in this study or did not focus on patients treated with BRAF-MEK inhibitors [13,14].

### Trials vs. real-world

Based on the baseline characteristics of real-world patients treated with first-line BRAF-MEK inhibitors, 53% (231/435) would have been considered ineligible for trials due to ECOG PS≥2 and the presence of brain metastases. In previous research, we showed that patients with brain metastases treated with BRAF-MEK inhibitors have similar outcomes to matched patients included in postapproval clinical trials [15]. The number of ineligible patients for phase III trials in this study is higher than reported in our previous study (40%), in which we focused on all patients with advanced melanoma, regardless of the treatment [16]. This difference can be explained by the generally poorer characteristics of patients treated with first-line BRAF-MEK inhibitors. Real-world patients with characteristics corresponding to the phase III trial inclusion criteria (eligible) had an mOS of 17.9 months and a 2-year survival rate of 39%, both lower than in the phase III trials. In pooled data from the COMBI-d and COMBI-v trial, the median OS was 25.9 months for dabrafenib/trametinib, with a 2-year survival rate of 52% [5]. In the coBRIM trial, this was 22.5 months and 49%, respectively [17].

Our real-world patients eligible for trial participation still had poor baseline characteristics, such as more often stage IV M1c disease and highly elevated LDH levels of >500 U/l (Supplemental Material 3, Supplemental digital content 1, http://links.lww.com/MR/A296). BRAF-MEK inhibitors are preferred as first-line treatment for patients with aggressive diseases because of their immediate effect. Patients with LDH ≥500 U/l at diagnosis almost did not reach long-term survival (<4%). This is in line with previously reported research which showed that LDH is a strong prognostic factor for survival in patients treated with BRAF inhibitor monotherapy [18]. Long-term survival does not solely depend on patient- and tumor characteristics but also on real-world treatment strategies. Real-world treatment can be different than the tightly controlled treatment setting in trials in terms of treatment duration, early discontinuation because of toxicity, compliance and other treatment regimes can contribute to differences in effectiveness.

Thirteen patients in the study cohort had a V600-BRAF mutation other than the V600E or V600K mutation. Analyses performed excluding these patients led to the same results (data not shown). Previous real-world research showed a statistically significant difference in PFS and OS for different V600 mutations [19]. In the real-world population, subsequent anticancer therapy was used in 52% of the patients, which was comparable to data from the COMBI-v and -d trial (53%) and the coBrim trial (51%) [5,17].

Female gender and ECOG PS ≤1 were positively associated with survival in the trials but were not significantly associated with OS in our multivariate Cox model [20]. Although patient characteristics were more favorable as survival improved, some patients reaching 3- and 4-year survival also had poor characteristics at baseline. In 62% of the 4-year survivors, the stage of disease was IVM1c, 32% LDH was elevated at baseline and 17% had brain metastases, compared to respectively 93, 54 and 48% in patients not reaching long-term survival (<2-year OS). In patients with LDH >500 U/l, first-line treatment with BRAF-MEK inhibitors continued until progression rarely resulted in long-term survival. This is in line with our previously published analysis of patients with LDH >500 U/l, in which we showed that induction treatment might lead to more favorable outcomes for these patients [21].

### Treatment strategies

A majority (65%) of the long-term survivors on first-line BRAF-MEK inhibitors received subsequent immunotherapy compared to 47% of the patients not reaching 2-year OS. Immunotherapy prolongs median survival, and subsequent immunotherapy is expected to contribute to long-term survival in our study population. Still, we cannot say which sequence is most valuable because comparing two groups would lead to confounding by indication as these treatment strategies are based on the characteristics of the patients and therefore a well-considered choice. We previously showed that in matched patients with BRAFV600-mutant advanced melanoma with relatively favorable characteristics, first-line anti-PD-1 monotherapy leads to an improved OS compared to first-line BRAF/MEK inhibition [22]. Recently reported prospective trial data from the randomized phase 3 Dreamseq trial comparing first-line ipilimumab/nivolumab vs. dabrafenib/trametinib confirmed this [23]. Similarly, data from SECOMBIT, a three-arm randomized phase 2 trial comparing first-line encorafenib/binimetinib, first-line ipilimumab/nivolumab and encorafenib/binimetinib induction followed by ipilimumab/nivolumab, thus far seem to favor first-line immune-checkpoint inhibitors [24].

### Limitations

The treatment landscape of patients with advanced melanoma changed over the years. This has resulted in more treatment options and improved survival. Ipilimumab/nivolumab combination therapy has been increasingly used since 2017. As a result, BRAF-MEK inhibitors have been prescribed to a lesser extent in the first line. Still, patients with a contraindication for immunotherapy can be treated with BRAF-MEK inhibitors, and these data are valuable for these patients.

We chose to investigate patients diagnosed between 2013 and 2017 to limit the time bias and to have sufficient follow-up time. However, novel treatments as subsequent therapies could still have influenced these results. Another limitation is the number of patients included in this study, resulting in a selected group of patients with mainly aggressive disease. Furthermore, our study population was not treated with encorafenib/binimetinib because these BRAF-MEK inhibitors gained market access after 2017. Our data can therefore not be extrapolated for these drugs.

### Conclusion

Survival rates of real-world patients with advanced melanoma treated with BRAF-MEK are lower than in trial patients, which is possibly related to poorer characteristics with regard to age, LDH level and metastatic sites. Still, patients with poor characteristics treated with first-line BRAF-MEK can achieve long-term survival, especially when obtaining a complete response.

## Acknowledgements

The real-world use and clinical outcomes of patients treated with BRAF-MEK inhibitors has not been previously researched. The lower survival rates of real-world patients can be explained by several factors. This information is important for daily clinical practice in the treatment choices for real-world patients.

The data that support the findings of this study are available from the Dutch Institute for Clinical Auditing (DICA). Restrictions apply to the availability of these data, which were used under license for this study. Data are available with the permission of DICA (www.dica.nl).

The Dutch Melanoma Treatment Registry (DMTR), the Dutch Institute for Clinical Auditing foundation, received a start-up grant from the governmental organization The Netherlands Organization for Health Research and Development (ZonMW, project number 836002002). The DMTR is structurally funded by Bristol Myers Squibb, Merck Sharpe & Dohme, Novartis, and Roche Pharma. Roche Pharma stopped funding in 2019, and Pierre Fabre started funding the DMTR in 2019. For this work, no funding was granted.

### Conflicts of interest

K.S. has advisory relationships with Bristol Myers Squibb, Novartis, MSD, Pierre Fabre, AbbVie and received honoraria from Novartis, MSD and Roche, all paid to institution. M.A. has advisory board/consultancy honoraria from Amgen, Bristol Myers Squibb, Novartis, MSD-Merck, Merck-Pfizer, Pierre Fabre, Sanofi, Astellas, Bayer. Research grants Merck-Pfizer. Not related to current work and paid to institute. C.B. has received commercial research grants from Novartis, Bristol Myers Squibb, and NanoString; is a paid advisory board member for Bristol Myers Squibb, MSD, Roche, Novartis, GlaxoSmithKline, AstraZeneca, Pfizer, Lilly, GenMab, and Pierre Fabre; and holds ownership interest in Uniti Cars, Neon Therapeutics, and Forty Seven. M.B.-S. has consultancy/advisory relationships with Pierre Fabre, MSD and Novartis. J-W.dG has consultancy/advisory relationships with Bristol Myers Squibb, Pierre Fabre, Servier, MSD, Novartis. J.H. has advisory relationships with Aimm, Achilles Therapeutics, Amgen, AstraZeneca, Bayer, Bristol Myers Squibb, BioNTech, GSK, Immunocore, Ipsen, MSD, Merck Serono, Molecular Partners, Novartis, Neogene Therapeutics, Pfizer, Roche/Genentech, Sanofi, Seattle Genetics, Third Rock Ventures, Vaximm and has received research grants not related to this paper from Amgen, Bristol Myers Squibb, MSD, BioNTech, Neogene Therapeutics and Novartis. All grants were paid to the institutions. G.H. consultancy/advisory relationships with Amgen, Bristol Myers Squibb, Roche, MSD, Pfizer, Novartis, Pierre Fabre and has received research grants not related to this article from Bristol Myers Squibb, Seerave and were paid to the institution. E.K. has consultancy/advisory relationships with Bristol Myers Squibb, Novartis, Merck, Pierre Fabre, Lilly and Bayer (all paid to institution), and received research grants not related to this article from Bristol Myers Squibb. R.v.R. has advisory board/ consultancy honoraria from Pfizer and an expert meeting fee from Roche. A.v.d.V. has consultancy relationships with Bristol Myers Squibb, MSD, Roche, Novartis, Pierre Fabre, Pfizer, Sanofi, Ipsen, Eisai, Merck. H.W. has received travel expenses from Ipsen and Astellas, and received speaker honoraria from Astellas and Roche. A.v.d.E. has advisory relationships with Amgen, Bristol Myers Squibb, Roche, Novartis, MSD, Pierre Fabre, Sanofi, Pfizer, Ipsen, Merck and has received research study grants not related to this article from Sanofi, Roche, Bristol Myers Squibb, Idera and TEVA and has received travel expenses from MSD Oncology, Roche, Pfizer and Sanofi and has received speaker honoraria from BMS and Novartis. For the remaining authors, there are no conflicts of interest.

## Supplementary Material


