# Supplementary material for: Long-term survival of patients with advanced melanoma treated with BRAF-MEK inhibitors
Source: Melanoma Res. 2022 Sep 5;32(6):460–8. doi: 10.1097/CMR.0000000000000832 (PMC9612708; doi:10.1097/CMR.0000000000000832)

Variable		N	Events	HR (CI)	P-value
**Age**					
	<50	84	62	Reference	
	50-59	92	73	1.00 (0.70, 1.42)	0.979
	60-69	94	81	1.23 (0.87, 1.73)	0.246
	>70	77	70	1.55 (1.08, 2.21)	0.016
**Gender**					
	Male	186	159	Reference	
	Female	161	127	0.90 (0.70, 1.15)	0.399
**Stage**					
	IIIc, IVm1a, IVm1b	25	12	Reference	
	IV-M1c	322	274	1.72 (0.91, 3.24)	0.095
**ECOG**					
	0	130	101	Reference	
	1	134	110	1.17 (0.89, 1.54)	0.265
	2-4	83	75	1.06 (0.76, 1.49)	0.726
**LDH**					
	Normal	165	126	Reference	
	250-500	114	94	1.14 (0.85, 1.53)	0.382
	>500	68	66	2.63 (1.82, 3.82)	<0.001
**Organsites**					
	<3	118	82	Reference	
	>2	229	204	1.54 (1.15, 2.07)	0.004
**Brainmetastasis**					
	No	200	152	Reference	
	Asymptomatic	48	43	1.47 (1.02, 2.10)	0.037
	Symptomatic	99	91	1.78 (1.32, 2.39)	<0.001
**Livermetastasis**					
	No	210	163	Reference	
	Yes	137	123	1.09 (0.81, 1.45)	0.576

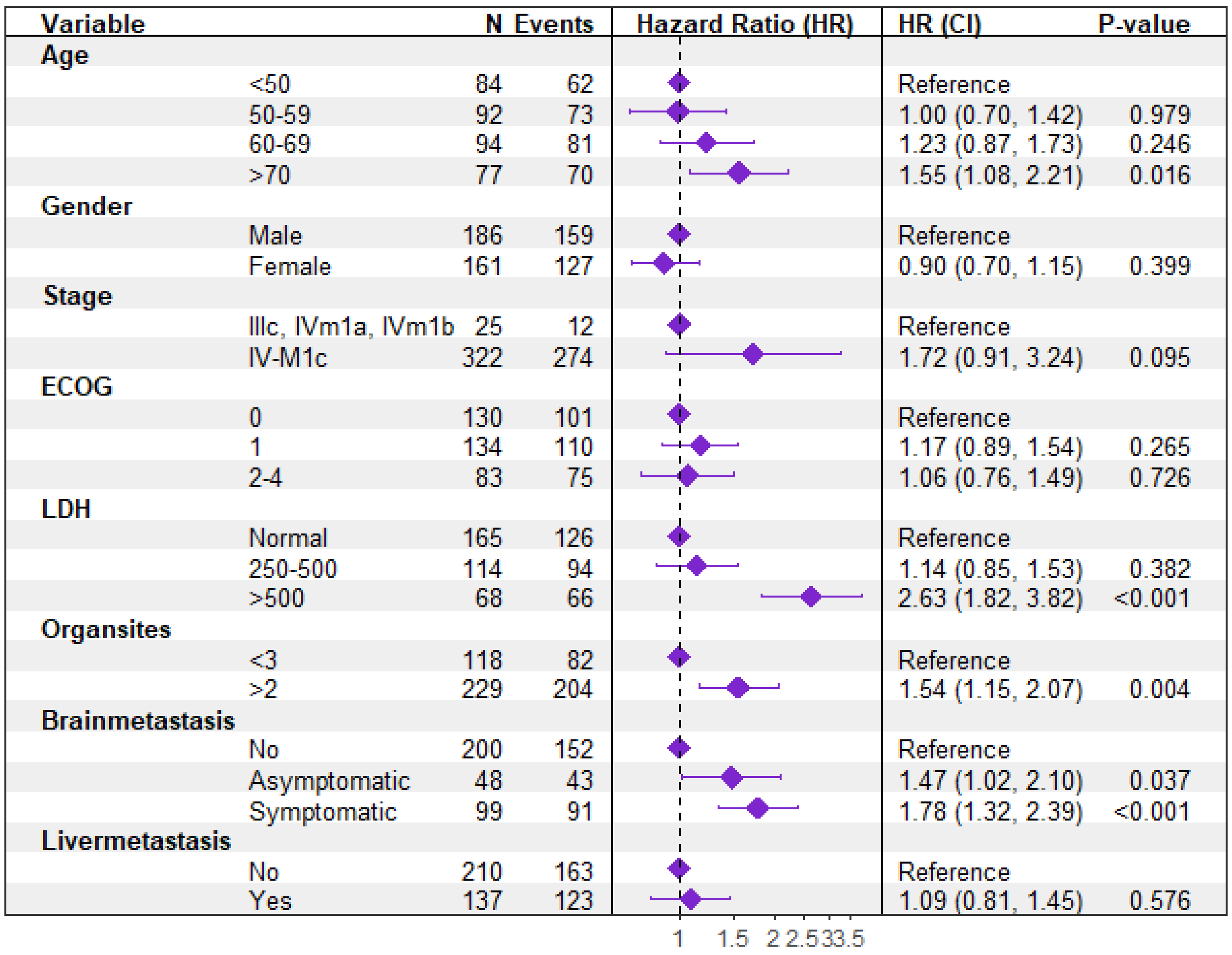

Supplement 1: Forest plot including multivariable Cox proportional hazard model for death within patients with advanced melanoma treated with first-line BRAF-MEK inhibitors.

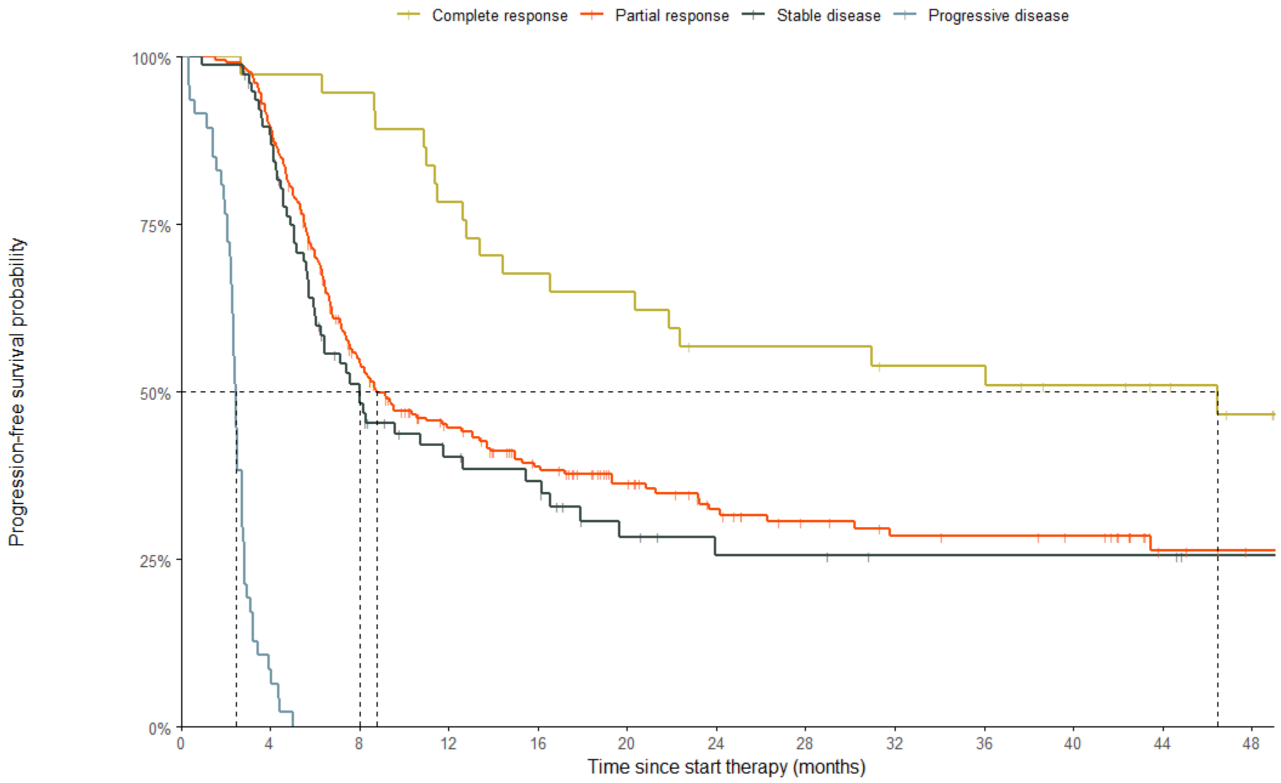

Number at risk

	0	4	8	12	16	20	24	28	32	36	40	44	48
Complete response	37	36	35	29	25	24	20	20	18	18	15	13	10
Partial response	257	230	126	89	69	52	39	30	26	25	23	11	8
Stable disease	78	68	34	23	20	12	9	9	7	7	7	7	5
Progressive disease	47	4	0	0	0	0	0	0	0	0	0	0	0

Time since start therapy (months)

Supplement 2: Kaplan-Meier estimates of median progression-free survival of patients with advanced melanoma treated with first-line BRAF-MEK inhibitors according to best-overall responses.

Fourteen patients were not evaluable for response, and two patients were excluded from this analysis because of missing data on progressive disease.

Supplement 3: Patient- and tumor characteristics of patients treated with first-line BRAF-MEK inhibitors who are considered eligible and ineligible for phase III trial participation.

	Eligible	Ineligible	P-value
**Patients; n**	208	227	
**Median age, year (range)**	60 (26-91)	59 (19-87)	0.981
**Age categories; n (%)**			0.583
**<50yr**	51 (24.5)	53 (23.3)	
**50-59yr**	52 (25.0)	65 (28.6)	
**60-69yr**	59 (28.4)	51 (22.5)	
**70-79yr**	33 (15.9)	44 (19.4)	
**>80yr**	13 (6.2)	14 (6.2)	
**Gender; n (%)**			0.701
**Male**	107 (51.4)	122 (53.7)	
**Female**	101 (48.6)	105 (46.3)	
**ECOG PS; n (%)**			
**0**	99 (47.6)	52 (22.9)	<0.001
**1**	87 (41.8)	64 (28.2)	
**≥2**	2 (1.0)	88 (38.8)	
**Unknown**	20 (9.6)	23 (10.1)	
**LDH level (U/l); n (%)**			0.103
**Normal**	102 (49.0)	107 (47.1)	
**250-499**	65 (31.2)	72 (31.7)	
**>500**	35 (16.8)	43 (19.0)	
**Not determined**	3 (1.4)	5 (2.2)	
**Stage (AJCC 7th); n (%)**			<0.001
**IIIc**	28 (13.5)	3 (1.3)	
**IV-M1a**	13 (6.2)	1 (0.4)	
**IV-M1b**	9 (4.3)	3 (1.3)	
**IV-M1c**	158 (76.0)	220 (96.9)	
**Metastasis in ≥3 organ sites; n (%)**			<0.001
**Yes**	105 (50.5)	166 (73.1)	
**No**	103 (49.5)	61 (26.9)	
**Brain metastasis; n (%)**			<0.001
**No**	171 (82.2)	48 (21.1)	
**Yes, asymptomatic**	0 (0.0)	54 (23.8)	

**Yes, symptomatic**	5 (2.4)	119 (52.4)	
**Unknown**	32 (15.4)	6 (2.6)	
**Liver metastasis; n (%)**	81 (38.9)	82 (36.1)	<0.001
**Best overall response; n (%)**			<0.001
**Complete response**	32 (15.4)	5 (2.2)	
**Partial response**	112 (53.8)	145 (63.9)	
**Stable disease**	39 (18.8)	39 (17.2)	
**Progressive disease**	22 (10.6)	27 (11.9)	
**LTFU**	3 (1.4)	11 (4.8)	
**BRAF mutation; n (%)**			0.002
**V600E**	177 (85.1)	184 (81.1)	
**V600K**	31 (14.9)	30 (13.2)	
**Other**	0	13 (5.7)	
**Subsequent therapy after first-line BRAF/MEK; n (%)**			0.388
**No**	87 (41.8)	105 (46.3)	
**Immunotherapy**	110 (52.9)	116 (51.1)	
**Other**	11 (5.3)	6 (2.6)	

Supplement 4: Therapy flow of long-term survivors who received BRAF-MEK inhibitors as first-line treatment

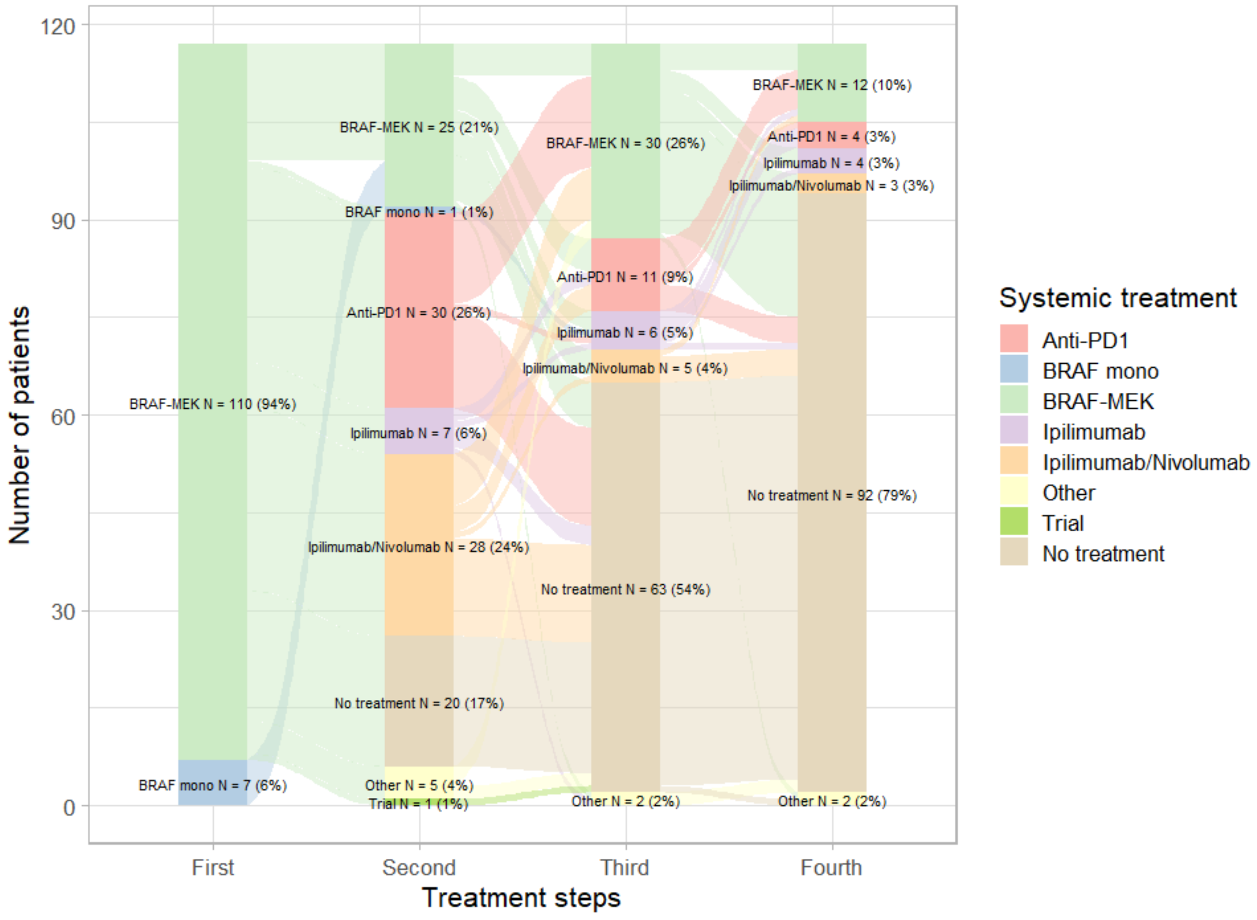

Supplement: Supplementary file 1 [file mr-32-460-s001.pdf]
